# Transient biopsy-proven progressive multifocal leukoencephalopathy-immune reconstitution inflammatory syndrome (PML-IRIS) in an elderly woman without known immunodeficiency: a case report

**DOI:** 10.1186/s12883-024-03945-0

**Published:** 2024-11-09

**Authors:** Susanne Gaarden Ingebrigtsen, Kristin Smistad Myrmel, Stian Henriksen, Gry Charlotte Wikran, Marit Herder, Garth D. Tylden, Hans H. Hirsch, Christine Hanssen Rinaldo

**Affiliations:** 1 Department of Neurology, University Hospital of North Norway, Tromsø, Norway; 2 Department of Pathology, University Hospital of North Norway, Tromsø, Norway; 3 Department of Microbiology and Infection Control, University Hospital of North Norway, Tromsø, Norway; 4 Metabolic and Renal Research Group, Department of Clinical Medicine, UiT The Arctic University of Norway, Tromsø, Norway; 5 Department of Radiology, University Hospital of North Norway, Tromsø, Norway; 6 Department of Medical Biology, UiT , The Arctic University of Norway, Tromsø, Norway; 7 Department of Virology, Norwegian Institute of Public Health, Oslo, Norway; 8 Division of Transplantation and Clinical Virology, Department of Biomedicine, Faculty of Medicine, University of Basel, Basel, Switzerland

**Keywords:** JC polyomavirus, Progressive multifocal leukoencephalopathy, Immune reconstitution inflammatory syndrome, MRI, Brain biopsy, Flow cytometric immunophenotyping, CD8 + T cells, Macrophages, JCPyV antibody, Case report

## Abstract

**Background:**

Progressive multifocal leukoencephalopathy (PML) is a severe opportunistic brain disease caused by lytic JC polyomavirus (JCPyV) replication in oligodendrocytes. Although JCPyV infection is common in the general population, PML almost exclusively occurs in patients immunocompromised due to untreated HIV/AIDS, haematological malignancies, primary immunodeficiencies, solid organ transplantation, or immunomodulatory treatment of autoimmune diseases. There is no effective antiviral treatment, and recovery depends on immune reconstitution. Paradoxically, initiation of antiretroviral therapy for HIV/AIDS or interruption of immunomodulating treatment can worsen the clinical manifestations due to immune reconstitution inflammatory syndrome (IRIS). Here, we report an unusual case of spontaneous IRIS in a 76-year-old immunocompetent woman, unmasking PML and leading to unexpected recovery.

**Case presentation:**

The patient was admitted to the hospital due to psychosis, speech impairment, and behavioral changes over the last three months. She had previously been healthy, except for a cerebellar stroke secondary to paroxysmal atrial fibrillation. Magnetic resonance imaging (MRI) revealed multiple contrast-enhancing white matter lesions suspicious of cancer metastases. Due to suspicion of edema, dexamethasone was administered, and the patient was released while waiting for a stereotactic brain biopsy. Eight days later, she suffered tonic seizures and was readmitted. Intravenous levetiracetam gave rapid effect, but the patient was paranoid and non-cooperative, and dexamethasone was unintentionally discontinued. Ten days later, the brain biopsy revealed demyelination, abundant perivascular T cells, macrophages, and scattered JCPyV-infected oligodendrocytes, rendering the diagnosis of PML-IRIS. The cerebrospinal fluid contained low amounts of JCPyV-DNA, and plasma contained high levels of anti-JCPyV immunoglobulin G. Despite extensive immunological testing, no evidence of immunodeficiency was found. The patient gradually recovered clinically and radiologically. More than 19 months after diagnosis, the patient has only a slight impairment in language and behavior.

**Conclusions:**

An apparently immunocompetent elderly person developed clinically symptomatic PML, which spontaneously resolved with symptoms and signs of IRIS. The atypical MRI lesions with contrast enhancement and the lack of known immunological risk factors for PML delayed the diagnosis, eventually proved by biopsy. PML and PML-IRIS should be considered in the differential diagnosis of patients presenting CNS symptoms and focal lesions with contrast enhancement on MRI.

## Background

Progressive multifocal leukoencephalopathy (PML) is a demyelinating encephalopathy caused by the replication of JC polyomavirus (JCPyV) in the myelin-producing oligodendrocytes [1–3]. Only rarely are the cerebellar granule neurons infected. The subsequent tissue damage may cause visual, motor, and/or cognitive impairments and is frequently fatal. JCPyV is a common virus. In European populations, the seroprevalence increases with age and peaks around 70 years when 50—80% have immunoglobulin G (IgG) against JCPyV major capsid protein 1 (Vp1) [4–8]. Primary JCPyV infection appears to be subclinical and leads to lifelong persistence in epithelial cells of the renourinary tract and possibly also in lymphocytes and bone marrow [9]. Episodes of viral reactivation with shedding in urine are common, as they have been reported in 19% of randomly collected urine samples from 400 healthy blood donors [5]. It is unclear if JCPyV enters the central nervous system (CNS) during primary infection or later during episodes of viral reactivation [10]. Still, PML predominantly affects patients with impaired cellular immunity. In the context of human immunodeficiency virus (HIV) infection, PML is viewed as an AIDS-defining disease as it is mainly seen with severe immunodeficiency, i.e., CD4 + T cell numbers below 200 per microlitre. Other immunosuppressive conditions or treatments associated with PML include primary immunodeficiencies, hematological disorders and malignancies and their therapy, organ or stem cell transplantation, and immunomodulatory therapy for autoimmune diseases [9]. For instance, PML can occur during treatment for relapsing–remitting multiple sclerosis (MS) with natalizumab. This monoclonal antibody prevents lymphocytes from migrating from the blood into the CNS, suppressing T-cell-mediated immune surveillance in the brain [11]. Some cases of PML in the context of only mild immunosuppression, and very rarely in immunocompetent patients, have also been described [12–16].

The JCPyV variants detected in brain tissue samples and cerebrospinal fluids (CSFs) from PML patients typically have genome rearrangements in the non-coding control region and specific point mutations in the VP1 gene [17–19]. While the genome rearrangements seem to increase the viral replication potential and facilitate neurotropism, the amino acid changes in Vp1 may lead to loss of receptor binding and escape from neutralizing antibodies, but this requires further studies [20–22].

Antiviral therapy for JCPyV is lacking, and PML treatment relies on re-establishing the immune function when possible. Paradoxically, reconstitution of pathogen-specific immune function following the initiation of HIV-suppressive antiretroviral therapy (ART) in patients with AIDS-related PML or discontinuation of immunomodulating agents like natalizumab in MS patients may lead to immune reconstitution inflammatory syndrome (IRIS) [10, 23, 24]. This adverse severe event usually aggravates the symptoms and pathology. Other pathogens reported to cause CNS-IRIS in the setting of HIV are cryptococcus neoformance, toxoplasma gondii, Mycobacterium tuberculosis, Candida organisms, cytomegalovirus (CMV), and varicella zoster virus (VZV) [23, 25].

Here, we describe the unique case of spontaneous IRIS unmasking PML in a 76-year-old immunocompetent woman. Accordingly, the diagnosis of PML was delayed. This case suggests that PML with or without spontaneous IRIS is more common in apparently immunocompetent elderly persons than previously perceived and should be considered in the differential diagnosis of patients presenting with CNS symptoms and signs.

## Case presentation

**Fig. 1:**
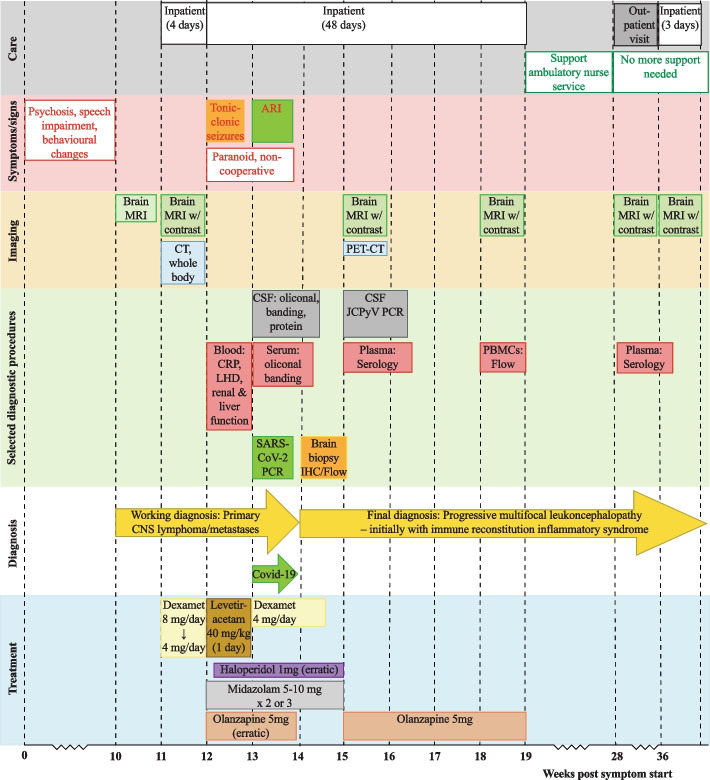
Timeline of symptoms and signs, selected diagnostic procedures, and treatment. On the X-axis, time is shown as weeks after the symptoms started

**Fig. 2:**
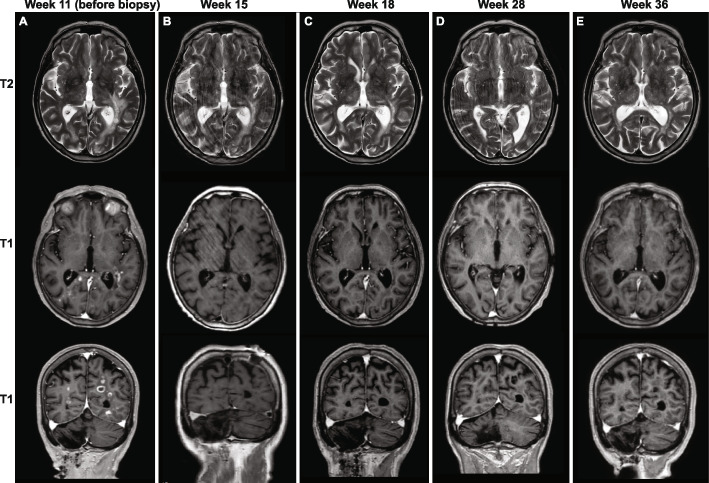
Chronological changes in brain magnetic resonance imaging (MRI). T2-flair on the top row and T1-flair on the next two rows. **A** 11 weeks after the symptoms started, i.e., 18 days before the biopsy, patchy T2 hyperintensities in the white matter are seen. The lesions are periventricular, mostly around the occipital- and temporal horns, with a left-sided predominance. There are also some subcortical lesions, nodular contrast enhancement in smaller lesions, and ring-like patterns in the more extensive lesions. The lesions are partly confluent with surrounding edema. **B** 15 weeks after the symptom started, i.e., 10 days after the biopsy, there is still some edema but almost complete regression of the contrast enhancement in the lesions. The patient was moving slightly. **C** 18 weeks after symptoms started, there were no or only minimal changes from the previous examination. **D** 28 weeks after symptoms started, there were no or only minimal changes from the previous examination. **E** 36 weeks after symptoms started, there were no or only minimal changes from the previous examination

A 76-year-old woman presented with psychosis, speech impairment, and behavioral changes of insidious onset over the last 10 weeks (Fig. 1). Ten years ago, she had a cerebellar stroke due to paroxysmal atrial fibrillation with right-sided abducens paralysis and slight balance impairment as sequelae. She was otherwise a healthy, immunocompetent non-smoker who lived at home with her spouse. She had no history of exotic travel or infectious disease events before presentation. On examination, she had a slight balance impairment, abducens paresis of the right eye, slurred speech, slightly impaired short-term memory, and acoustic and visual hallucinations. Neurological findings were otherwise normal. Her general practitioner referred her to magnetic resonance imaging (MRI) of the brain, which revealed multiple white matter lesions, raising suspicion of cancer metastases. She was admitted to the oncology clinic for further examination. An MRI with intravenous contrast revealed patchy T2 hyperintensities in the white matter (Fig. 2 A). Numerous lesions were periventricular, mainly around the occipital- and temporal horns, with a left-sided predominance. There were also some subcortical lesions. Nodular contrast enhancement was seen in smaller lesions and ring-like patterns in larger lesions. The lesions were partly confluent with surrounding edema.

Moreover, MR spectroscopy demonstrated an increased choline/creatinine ratio, a moderately reduced N-acetylaspartate, and a lactate/lipid peak. The image findings were first interpreted as primary CNS lymphoma or multiple cancer metastases of unknown origin.

Miliary tuberculosis, neurosarcoidosis, and angiocentric lymphoma were also mentioned as possible differential diagnoses. A whole-body computerized tomography (CT) scan was performed but showed no evidence of underlying occult malignancies. Ultrasound of the uterus and ovaries and cervical histology were normal. A positron emission tomography-CT and stereotactic brain biopsy were planned. Until then, the patient was discharged to her home with dexamethasone 8 mg daily to reduce the edema related to the MRI lesions.

After four days of dexamethasone treatment, her symptoms were reported as improving, and the dexamethasone dosage was reduced to 4 mg daily. Four days later, i.e., about 12 weeks after the symptoms started, she was acutely admitted to the hospital due to tonic seizures. At admission, she was unconscious with a Glasgow coma scale score of 10 and jaw lock. She was treated with Levetiracetam 40 mg/kg intravenous with effect, and later, on the same day, the treatment was discontinued after an electroencephalogram (EEG) without epileptogenic traits. However, the patient was paranoid and non-cooperative and refused intake of most medications for almost two weeks, except midazolam 5–10 mg mixed into soft drinks 2–3 times daily. No new MRI was conducted due to a lack of cooperation. C-reactive protein (CRP), renal and liver function, B12, folate, and LDH were in the normal range.

During the first week of hospitalization, i.e., week 13 after the symptoms started, her CSF only showed slightly elevated protein 580 mg/L [ref. 150—450 mg/L] and albumin 363 mg/L [ref. 100 – 300 mg/L], and a cell count less than 4/µL. Moreover, the CSF showed seven oligoclonal bands, whereas only two could be seen in serum, indicating intrathecal IgG synthesis. The albumin index in CSF was 10 (ref. < 9), i.e., slightly elevated and suggested a minor blood–brain barrier leakage. Immunophenotyping of CSF cytology showed normal morphology, few lymphocytes, and a normal CD4 + /CD8 + ratio of 1.2, and negative results for acid-fast bacilli and mycobacterial culture. Three days later, the patient developed flu-like symptoms such as fever, runny nose, and a light cough. Her spouse was also sick and had visited her in the hospital just prior. SARS-CoV-2 (Omicron sub-lineage BQ1.13.1) was detected with high viral loads. The infection was clinically mild, although her CRP peaked at 50 mg/L, and she recovered within less than a week without supportive treatment. After this, a significant clinical improvement was observed.

**Fig. 3:**
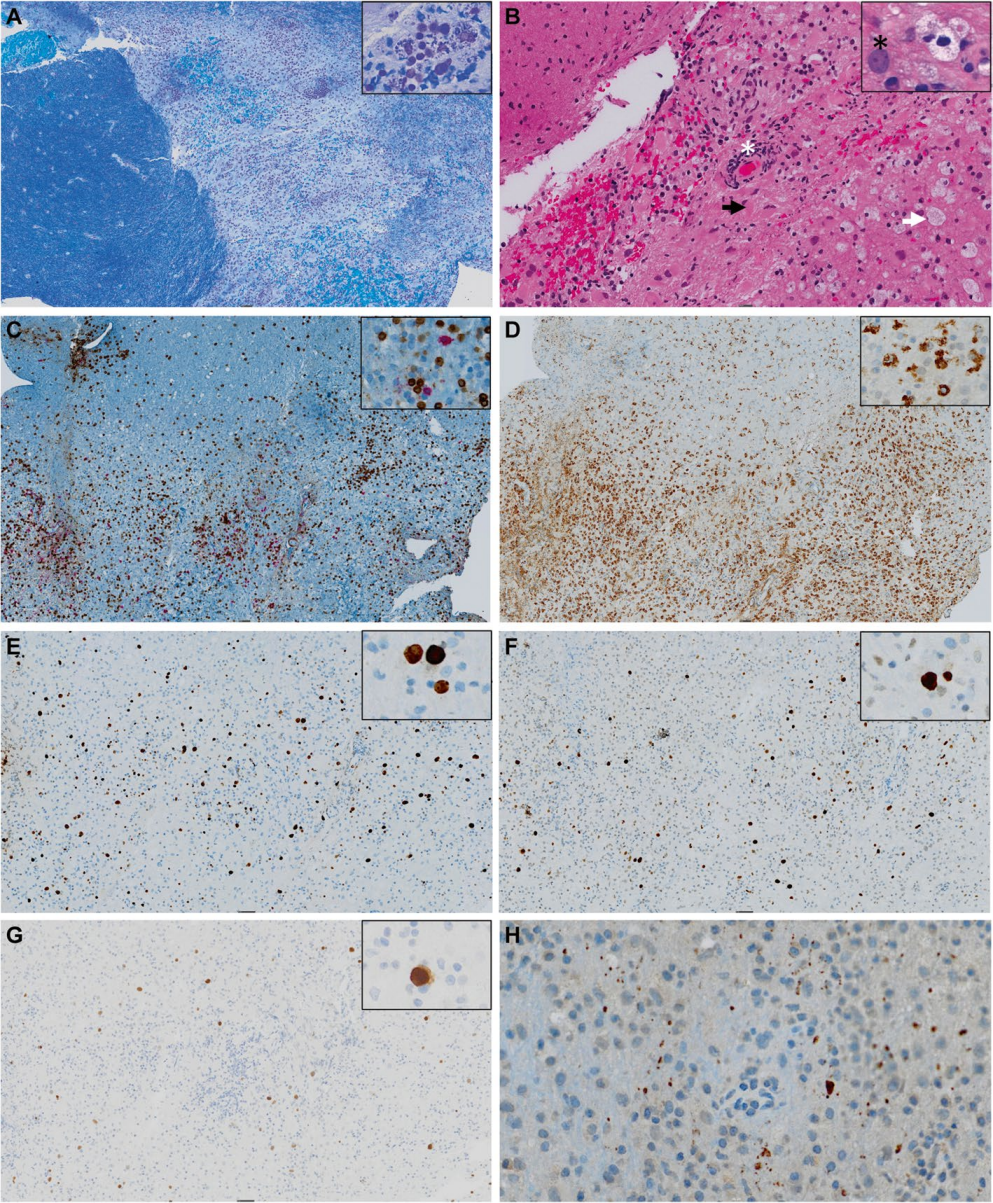
Staining and immunohistochemistry of the brain biopsy. **A** Luxol fast blue staining shows demyelination in the border of the grey and white matter junction. The insert shows macrophages containing myelin. **B** Hematoxylin and Eosin staining showing the presence of extensive inflammatory infiltrates with perivascular lymphoid cuffing (white asterisk), foamy macrophages (white arrow), and reactive astrocytes (black arrow). In the insert, an oligodendrocyte with enlarged nuclei is seen (black asterisk). **C** CD3 and CD20 immunostaining showing T-cells (in brown) and B-cells (in red), respectively. More T than B-cells are present. **D** CD68 immunostaining shows plenty of macrophages. **E** ki67 immunostaining shows strong staining of enlarged oligodendrocytes and other cells. **F** p53 immunostaining shows strong staining of enlarged oligodendrocytes and other cells. **G** JCPyV large T-antigen immunostaining, using the cross-reacting SV40 antibody Pab416, shows scattered enlarged oligodendrocytes with nuclear staining. **H** JCPyV Vp1 immunostaining (ab34756) shows grainy cytoplasmic staining. Magnification for panels **A**, **C**, **D**, **E**, **F**, and **G**: overview 5x, insert 20x. Magnification for panel **B**: overview 10x, insert 40x, and for panel **H**: 20x

About 14 weeks after the symptoms started, a stereotactic brain biopsy from the left parietal lobe was performed. Light microscopy of Luxol fast blue and hematoxylin/eosin stained sections revealed demyelination at the grey and white matter junction, with extensive inflammatory infiltrates, foamy macrophages, and prominent perivascular lymphoid cuffing (Fig. 3A and B). The transition to normal brain tissue was abrupt. On this background, scattered enlarged oligodendrocyte nuclei containing purple inclusions and large atypical astrocytes were seen (Fig. 3B). The astrocytes were presumed to be reactive, as no mitotic activity, endothelial proliferation, or atypia consistent with glioma was detected. Immunostaining showed that the lymphocytes were predominantly CD3 + T-cells, with only a few CD20 + B-cells (Fig. 3C), and confirmed the presence of abundant CD68 + macrophages (Fig. 3D). Several macrophages contained myelin in the cytoplasm, as shown by the Luxol fast blue staining (Fig. 3A). Neurofilament staining revealed spared axons in the demyelinated areas (not shown). As previously reported for JCPyV-infected oligodendrocytes and reactive astrocytes [26–28], prominent immunoreactivity for ki-67 (Fig. 3E) and p53 (Fig. 3J) was observed. Moreover, the SV40 large T-antigen antibody Pab416, which cross-reacts with JCPyV large T-antigen, labelled the nuclei in the enlarged oligodendrocytes (Fig. 3G). Of note, immunostaining failed to demonstrate Vp1 in the enlarged oligodendrocytes. Three different Vp1 antibodies that all stained control sections were tried. These included a cross-reacting rabbit polyclonal antiserum against the closely related BK polyomavirus (BKPyV) [18], a mouse monoclonal anti-JCPyV Vp1 antibody (ab34756) [29], and a rabbit anti-SV40 Vp1 serum (ab 53,977) [30]. However, some grainy cytoplasmic staining of cells in the lesion was observed (Fig. 3H), possibly suggesting that macrophages and reactive astrocytes contained Vp1. JCPyV proteins in macrophages have previously been described, including in a patient with a burnt-out PML lesion [27, 31]. Moreover, gram staining and staining for Epstein-Barr virus, human herpesvirus 8, and herpes simplex virus 1 (HSV-1) were negative (results not shown). Furthermore, flow cytometric immunophenotyping of the brain tissue confirmed increased lymphocytes and revealed that more than 90% of the lymphocytes were T-cells with a reduced CD4 + /CD8 + ratio of 0.5. Taken together, the results from the brain biopsy led to the diagnosis of PML and suggested the diagnosis of PML-IRIS.

**Fig. 4:**
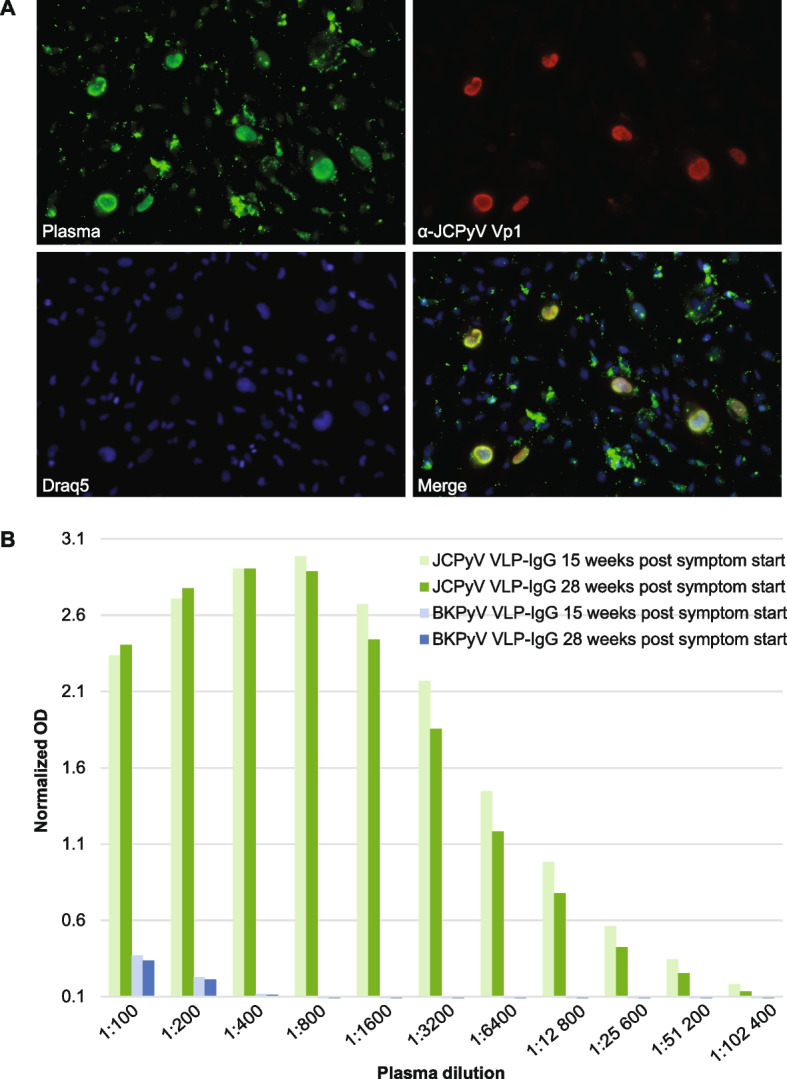
Serology JCPyV and BKPyV. **A** SVG-A cells were infected by JCPyV and fixed by methanol before immunofluorescence staining was performed using a combination of patient plasma from 15 weeks post symptom start and the mouse monoclonal anti-JCPyV Vp1 antibody (ab34756). Alexa 488-labelled goat-anti human IgG (green) and Alexa 568-labelled goat anti-mouse IgG (red) were used as secondary antibodies. Nuclei were labelled by Draq5 (blue). The plasma and the specific JCPyV Vp1 antibody show overlapping nuclear staining, suggesting that the patient has developed JCPyV Vp1 IgG. **B** JCPyV- and BKPyV-specific IgG using Vp1 virus-like particle (VLP)-based ELISAs. The two available serum samples were tested in several dilutions, and the result was normalized to a laboratory reference serum. The cut-off of the assay is 0.1. The plasma contained very high levels of JCPyV Vp1 IgG, as it could be diluted more than 100,000 times and still be positive. In contrast, it only contained low levels of BKPyV Vp1 IgG, which could only be detected with dilutions less than 800

Ten days after the biopsy, i.e., week 15 after the symptoms started, an MRI showed some edema but almost complete regression of the contrast enhancement in the lesions (Fig. 2B). Antipsychotic treatment with haloperidol was converted to a low dosage of olanzapine. Two days later, a new lumbar puncture was performed, and this time, analysis for HSV-1/2 DNA, VZV DNA, CMV DNA and JCPyV DNA, enterovirus RNA, and cryptococcus antigen but also aerobic and anaerobic cultures, fungus, total-tau, phospho-tau, beta-amyloid and protein 14–3-3 were included. A low copy number of JCPyV-DNA (about 10 copies/mL) [32] was detected, while all other analyses were negative. In plasma, JCPyV-specific IgG was detected by indirect immunofluorescence staining using JCPyV-infected SVG-A cells [33] (Fig. 4A).

A normalized JCPyV VLP-IgG ELISA [34] revealed high anti-JCPyV-IgG levels (nOD 2.7) (Fig. 4B), while a normalized BKPyV VLP-IgG ELISA [34] showed only low anti-BKPyV-IgG levels (nOD 0.23), both at plasma dilution 1:200 (Fig. 4B). Serologic tests for HIV-1/2, hepatitis B virus, hepatitis C virus, treponema pallidum, borrelia burgdorferi IgG, toxoplasma gondii IgG, and parvovirus B19, were all negative, as was the TB-QuantiFERON-IGRA test. Finally, IgG antibodies for HSV-1/2, VZV, Epstein Barr virus nuclear antigen, and CMV were positive. Only IgM for CMV was weakly positive, but the serological profile was interpreted as consistent with past primary CMV infection. Taken together, the biopsy results and the diagnostic virology indicated that the patient recently had experienced high-level JCPyV replication with corresponding brain pathology, to which she mounted a strong immune response. There was no indication that other pathogens were involved.

Four weeks after the brain biopsy, i.e., week 18 after the symptoms started, a follow-up MRI was performed. It showed minimal changes compared to three weeks earlier (Fig. 2C). An extensive workup was performed to assess whether the patient might have an underlying immunodeficiency. Phenotypic characterization of peripheral B and T lymphocytes by flow cytometry showed a normal CD4 + /CD8 + ratio of 2.4, but CD3 + and CD8 + T cells were slightly below the normal reference interval. There was no family- or personal history suggestive of immunodeficiency, and she was HIV-negative. Serum electrophoresis showed IgG 10.3 (7.00–16.00 g/L), IgA 3.14 (0.70–4.00 g/L), and IgM 1.7 (0.4–2.3 g/L). IgG subclasses 1, 2, and 4 were also normal, but she had a polyclonal increase of IgG subclass 3 of 1.15 g/L (0.11–0.85 g/L), which could fit with a severe bacterial or viral infection. Five weeks after the biopsy, i.e., 19 weeks after the symptoms started, the patient was discharged to her home with support from ambulatory nurse services.

A second follow-up examination was performed 28 weeks after the symptoms started. The MRI revealed no or minimal changes from the last examination (Fig. 2D). The CD4 + /CD8 + ratio in blood was normal at 2.66 with CD3 + and CD8 + T cells within the normal reference interval, and the level of anti-JCPyV Vp1 IgG was slightly reduced compared to 10 weeks earlier (Fig. 4B). Slight cognitive impairment persisted, but the patient was no longer in need of nursing assistance.

The last MRI was taken 36 weeks after the symptoms started (Fig. 2E) and was unchanged. At the last follow-up, about 18 months after the biopsy-based diagnosis of PML-IRIS, the patient was still doing well and lived at home.

## Discussion and conclusions

Although our patient did not have any of the well-known risk factors of PML or IRIS, PML-IRIS was diagnosed by the combined clinical, radiological, and pathological examinations. The inflammation seemingly unmasked PML and led to spontaneous recovery. The PML diagnosis was based on a brain biopsy, which is the gold standard for diagnosing PML. The biopsy revealed demyelination, JCPyV-infected oligodendrocytes (Pab416 positive cells), reactive astrocytes, and macrophages, and the latter cells contained phagocytized myelin and possibly also Vp1. The PML diagnosis was further supported by the finding of JCPyV-DNA in the CSF. At first admission (week 11 post-symptom start), axial MRI imaging showed multiple lesions with contrast enhancement. While MRI images of classic PML lesions rarely show marked contrast enhancement, this is commonly observed in PML patients with IRIS associated with interrupted natalizumab treatment of MS [35] and has also been described in PML patients undergoing adoptive transfer of JCPyV-specific T cells [36] and HIV/AIDS patients after initiation of ART [37]. The PML-IRIS diagnosis lacks a consensus definition but is normally used for clinical worsening due to inflammation [9]. The diagnosis was based on clinical worsening, MRI contrast enhancement, and the detection of many T-cells, particularly CD8^+^ T-cells, surrounding vessels, and dispersed in the brain tissue. Notably, infection with other pathogens reported to cause CNS-IRIS in HIV-positive patients, i.e., CMV, VZV, HIV, TB, toxoplasma gondii, Candida organisms, and cryptcoccus neoformans [23, 25], was excluded. That our patient had near-normal immunophenotyping, rapidly recovered from COVID-19, had a very high level of JCPyV-VLP-specific IgG, and otherwise normal antibody levels indicates that she was immunocompetent at the time of diagnosis and could mount a strong immune response. Our patient had only one instance of tonic seizures, and this was 8 days after the MRI detected pathologic contrast enhancement. Seizures are sometimes seen in PML patients but seem to be more common in patients with PML-IRIS [10, 38, 39]. Possibly, the strong immune response and inflammation led to cortical irritation and tonic seizures despite 8 days of dexamethasone treatment. To our knowledge, only one previous case of spontaneous IRIS associated with PML has been reported [40]. The 68-year-old man, without apparent immunodeficiency, did, however, not have a transient course but died about 4 months after the first admission.

What could have caused PML in our evidently immunocompetent patient? PML seems to arise mainly when cellular immunity is impaired. Interestingly, a study of HIV-positive and HIV-negative men showed that in contrast to renourinary BKPyV replication, renourinary JCPyV replication did not increase with decreased immune control [41], but this may be different in JCPyV-infected lymphocytes and bone marrow. Possibly, JCPyV reactivated in our patient during an unnoticed period of transient immune suppression, as previously suggested for an immunocompetent patient with fatal PML [42]. An alternative explanation is that a primary JCPyV infection caused PML. As shown in an extensive study of more than 800 MS patients between 19 and 59 years old receiving natalizumab, the annual JCPyV seroconversion rate is about 2% [43], much of which likely represents primary infection. Moreover, as known for a long time for influenza disease and more recently demonstrated by COVID-19, aging affects the immune system and makes many infections more severe in older individuals [44]. Since a primary JCPyV infection is believed to be symptomless, this would probably have gone unnoticed. There is at least one report on PML caused by JCPyV primary infection, but this was in a five-year-old girl suffering from severe combined immunodeficiency syndrome [45]. As we did not have earlier blood samples from our patient, we could not determine when JCPyV infected the patient. The patient also showed low seropositivity for BKPyV. Due to sequence homology, BKPyV-specific T cells may also recognize JCPyV peptides [10, 46, 47] and may have contributed to the resolution of PML. Possibly, the sudden paranoia and non-cooperative behavior during the first two weeks post-seizure was an adverse effect of the dexamethasone treatment [48].

Using the frequently utilized Pab416 antibody for JCPyV immunohistochemistry, scattered enlarged oligodendrocytes with positive staining for large T-antigen were seen. The relatively low number of infected cells observed is typically for PML-IRIS [38] and probably reflects the ongoing immune response. Usually, the enlarged oligodendrocytes in PML patients contain viral particles [9], but we could not detect nuclear Vp1 staining. As patients with PML may have JCPyV with mutations in the Vp1 gene [18], failed Vp1 staining may have been caused by amino acid changes in the Vp1 protein. Unfortunately, no more biopsy material was left for DNA sequencing of the viral genome. Our finding supports the diagnostic practice of immunohistochemical staining of brain biopsies for large T-antigen.

Diagnosing PML typically requires a combination of clinical suspicion, classical imaging, positive JCPyV PCR of CFS, and the gold standard, which is the detection of JCPyV DNA or proteins in a brain biopsy. In our case, the diagnosis was hampered by atypical findings on MRI, thus leading the radiologist to suggest other differential diagnoses. We speculate that IRIS led to a transient worsening of the clinical symptoms, which unmasked PML. We also think that IRIS contributed to the spontaneous recovery. This case highlights that PML may be more common in apparently immunocompetent elderly persons than previously perceived and that a contrast enhancement combined with clinical signs of PML should raise the suspicion of PML-IRIS.

## Data Availability

No datasets were generated or analysed during the current study.

## Abbreviations

ART: Antiretroviral therapy
BKPyV: BK Polyomavirus
CMV: Cytomegalovirus
CNS: Central nervous system
CRP: C-reactive protein
CSF: Cerebrospinal fluid
CT: Computerized tomography
EEG: Electroencephalogram
HIV: Human immunodeficiency virus
HSV: Herpes simplex virus
IgG: Immunoglobulin G
IRIS: Immune reconstitution inflammatory syndrome
JCPyV: JC Polyomavirus
MRI: Magnetic resonance imaging
MS: Multiple sclerosis
PML: Progressive multifocal leukoencephalopathy
TB: Tuberculosis
VZV: Varicella zoster virus
